# Supplementary material for: Effects of Complete and Partial Loss of the 24S-Hydroxycholesterol-Generating Enzyme Cyp46a1 on Behavior and Hippocampal Transcription in Mouse
Source: Biomolecules. 2024 Feb 21;14(3):254. doi: 10.3390/biom14030254 (PMC10968171; doi:10.3390/biom14030254)
Supplement: Supplementary file 1 [file biomolecules-14-00254-s001.zip › biomolecules-2840832-supplementary.pdf]

Supplementary materials.

Effects of complete and partial loss of the 24S-hydroxycholesterol-generating enzyme *Cyp46a1* on behavior and hippocampal transcription in mouse

Hong-Jin Shu[1*], Luke H. Ziolkowski[1,3*], Sofia V. Salvatore[1], Ann M. Benz[1], David F. Wozniak[1,2], Carla M. Yuede[1], Steven M. Paul[1,2], Charles F. Zorumski[1,2], Steven Mennerick[1,2]

[*]Contributed equally

[1]Department of Psychiatry

[2]Taylor Family Institute for Innovative Psychiatry Research

Washington University in St. Louis School of Medicine

[3] Current address: Department of Cellular and Molecular Physiology, Yale University School of Medicine

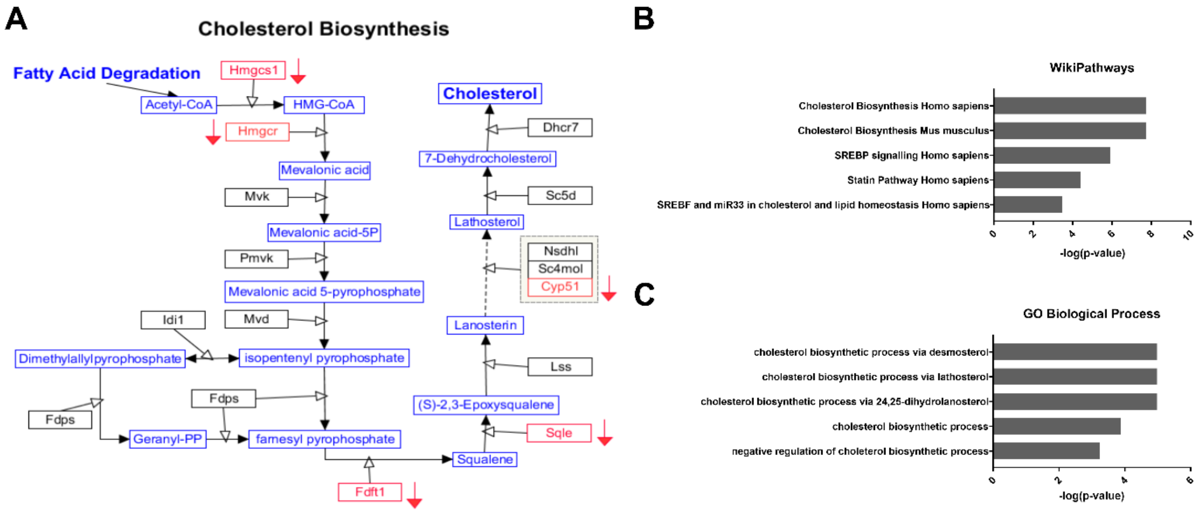

Supplemental Figure S1. Pathway analysis of transcriptional changes in hippocampal CA1 region. Red transcripts were downregulated in *Cyp46a1-/-* mice.

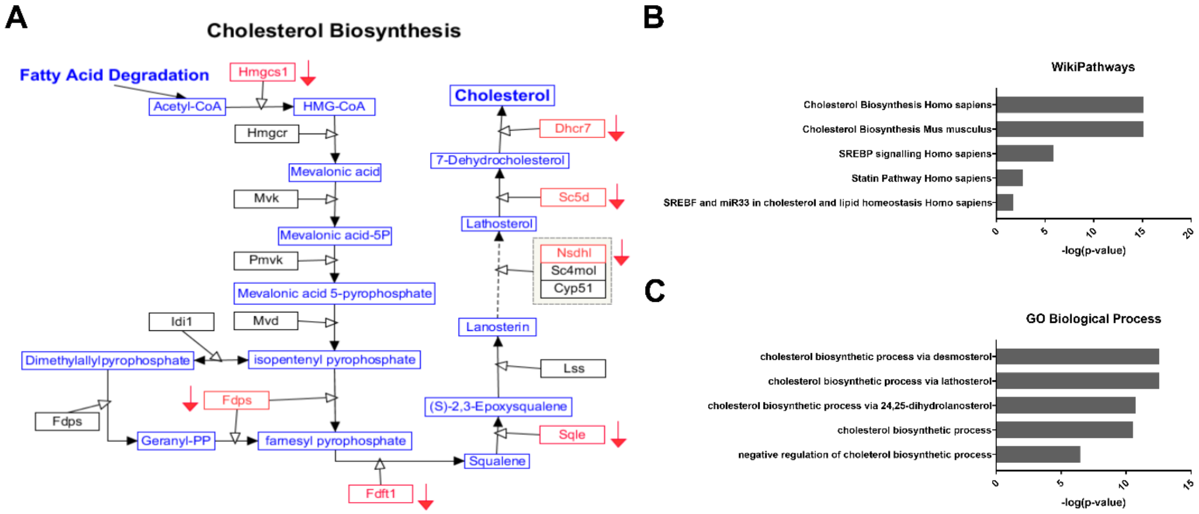

Supplemental Figure S2. Pathway analysis of transcriptional changes in hippocampal dentate gyrus region. Red transcripts were downregulated in *Cyp46a1-/-* mice.

	PND100		PND400
	KO	cKO	KO
24S-HC	----	---	
Locomotion	--	NC	NC
Conditioned fear freezing	++	NC	++
Morris maze acquisition	-		
Morris maze retention	NC		
LXR transcription targets	++	+	++

Supplemental Table S1. Summary of behavioral and transcriptional results from this study.

– and + designations indicate relative changes from WT mice. NC = no change. Morris water maze results were not evaluated in cKO or P400 animals because of lack of memory retention differences at P100 in KO animals.
